# Supplementary material for: Investigating the feasibility and potential of combining industry AMR monitoring systems: a comparison with WHO GLASS
Source: Wellcome Open Res. 2024 Oct 2;9:248. Originally published 2024 May 10. [Version 2] doi: 10.12688/wellcomeopenres.21181.2 (PMC11452768; doi:10.12688/wellcomeopenres.21181.2)
Supplement: Peer review history

# Reviewer report: Reviewer response for version 2

**DOI:** 10.21956/wellcomeopenres.25510.r102666

**Published:** 2024-10-04

**Authors:** Marcelo Pillonetto

**Affiliations:** 1 Pontifical Catholic University of Paraná, Curitiba, Brazil; 2 Molecular Bacteriology, Laboratório Central de Saúde Pública do Paraná - LACENPR, Curitiba, PARANÁ, Brazil

The authors have reviewed and adequately answered all the questions, comments, and suggestions raised by this reviewer.

Is the work clearly and accurately presented and does it cite the current literature?

Yes

If applicable, is the statistical analysis and its interpretation appropriate?

I cannot comment. A qualified statistician is required.

Are all the source data underlying the results available to ensure full reproducibility?

Partly

Is the study design appropriate and is the work technically sound?

Yes

Are the conclusions drawn adequately supported by the results?

Yes

Are sufficient details of methods and analysis provided to allow replication by others?

Yes

Reviewer Expertise:

Clinical Microbiology; Antimicrobial Resistance; AMR Surveillance; Bacterial Identification; Clinical Bacteriology Genomics; Public Health Microbiology; Phytobacter taxonomy.

I confirm that I have read this submission and believe that I have an appropriate level of expertise to confirm that it is of an acceptable scientific standard.

# Reviewer report: Reviewer response for version 1

**DOI:** 10.21956/wellcomeopenres.23426.r84308

**Published:** 2024-08-02

**Authors:** Marcelo Pillonetto

**Affiliations:** 1 Pontifical Catholic University of Paraná, Curitiba, Brazil; 2 Molecular Bacteriology, Laboratório Central de Saúde Pública do Paraná - LACENPR, Curitiba, PARANÁ, Brazil

The manuscript is a comprehensive study that combines and compares data from industry AMR monitoring and the WHO GLASS system. It points out some important limitations, such as the lack of distinction between isolates from hospital and community-acquired infections.

 It is a very well-written document about an urgent public health problem (AMR surveillance) that needs to be discussed globally.

 But some reviewing needs to be done before Indexing.

 1- In the Methods Section - Item *Data reformatting and combination*: 

 the authors affirm that   "...as previous work has suggested, resistance profiles are similar for commensal opportunistic pathogens across different sample sources ^15^."  This affirmation is very debatable since other studies have shown differences according to the sample sources. Especially if you consider that blood samples are much more common for inpatients and urine samples for outpatients.

 I suggest the authors broaden this subject, referencing the counterpart or include other references supporting the above affirmation.

 2- In the Item *Estimates of resistance across monitoring systems*

 Please correct *K. pneumonia *to *K. pneumoniae.*

 3—In Figure 2, there are some discrepancies that need to be better discussed about the possible results, especially the **drop in resistance to ceftriaxone in *E. coli* and *K. pneumoniae *from 2018 to 2019. Also, levofloxacin oscillation in *K. pneumoniae *and Ampicillin decreased in *E.  coli.*

 4- In the Discussion- *Summary*

 The authors affirm that *"*The GLASS dataset also does not report the type of pneumococcal infection..."

 In fact, up to 2023, the GLASS dataset only receives data from sepsis (non-meningitis) infections by *S. pneumoniae. *Please correct this affirmation.

 5- In the Data Availability section, the link https://searchamr.vivli.org/. is not accessing any web page.

Is the work clearly and accurately presented and does it cite the current literature?

Yes

If applicable, is the statistical analysis and its interpretation appropriate?

I cannot comment. A qualified statistician is required.

Are all the source data underlying the results available to ensure full reproducibility?

Partly

Is the study design appropriate and is the work technically sound?

Yes

Are the conclusions drawn adequately supported by the results?

Yes

Are sufficient details of methods and analysis provided to allow replication by others?

Yes

Reviewer Expertise:

Clinical Microbiology; Antimicrobial Resistance; AMR Surveillance; Bacterial Identification; Clinical Bacteriology Genomics; Public Health Microbiology; Phytobacter taxonomy.

I confirm that I have read this submission and believe that I have an appropriate level of expertise to confirm that it is of an acceptable scientific standard, however I have significant reservations, as outlined above.

## References

[1] : The Experience of Implementing a National Antimicrobial Resistance Surveillance System in Brazil. *Front Public Health* .2020;8: 10.3389/fpubh.2020.575536 575536 10.3389/fpubh.2020.575536 33520909 PMC7841397

# Response

**Published:** 2024-09-24

**Authors:** Quentin Leclerc

**Affiliations:** Institut Pasteur, Paris, Île-de-France, France

The manuscript is a comprehensive study that combines and compares data from industry AMR monitoring and the WHO GLASS system. It points out some important limitations, such as the lack of distinction between isolates from hospital and community-acquired infections. It is a very well-written document about an urgent public health problem (AMR surveillance) that needs to be discussed globally. But some reviewing needs to be done before Indexing.

 ​​​​​ **Response:** *Many thanks for your positive comments on our manuscript, and for your thoughtful review of our work. Please find below our point-by-point responses to your comments.*  

 1- In the Methods Section - Item Data reformatting and combination: the authors affirm that   "...as previous work has suggested, resistance profiles are similar for commensal opportunistic pathogens across different sample sources15."  This affirmation is very debatable since other studies have shown differences according to the sample sources. Especially if you consider that blood samples are much more common for inpatients and urine samples for outpatients. I suggest the authors broaden this subject, referencing the counterpart or include other references supporting the above affirmation.

**Response:** *We agree that this is a debatable subject, and that surprisingly little is known about differences according to sample sources. We have rephrased the sentence to clarify this uncertainty and state that this is an assumption we have made in our analysis also due to substantial lack of harmonisation across datasets. We have added a paragraph at the end of the Discussion to mention this important point as a possible next step of analysis which could be done using these industry monitoring systems, with further references (references 23-27 in the updated manuscript).*  

 2- In the Item Estimates of resistance across monitoring systems Please correct K. pneumonia to K. pneumoniae.

**Response:** *Thank you for spotting this mistake, which we have now corrected.*  

 3—In Figure 2, there are some discrepancies that need to be better discussed about the possible results, especially the drop in resistance to ceftriaxone in E. coli and K. pneumoniae from 2018 to 2019. Also, levofloxacin oscillation in K. pneumoniae and Ampicillin decreased in E.  coli.

**Response:** *We have added new sentences describing these trends in the Results section. Briefly, we believe several changes in resistance could be linked to a change in susceptibility testing volume, such as ceftriaxone resistance. We have added a new Supplementary Figure 2 to illustrate changes in testing volumes. For ampicillin however, we could not find a straightforward explanation, and have listed this as a point for future investigation.*  

 4- In the Discussion- Summary The authors affirm that "The GLASS dataset also does not report the type of pneumococcal infection..." In fact, up to 2023, the GLASS dataset only receives data from sepsis (non-meningitis) infections by S. pneumoniae. Please correct this affirmation.

**Response:** *Thank you for this information, we have corrected this sentence.*  

 5- In the Data Availability section, the link  https://searchamr.vivli.org/. is not accessing any web page.

**Response:** *Apologies for the inconvenience. We have confirmed that this link is functional, but unfortunately it can be sometimes necessary to refresh the page for it to be displayed properly. This is a server error which we cannot control, but we have added a sentence in the Data Availability section to inform readers.*

# Reviewer report: Reviewer response for version 1

**DOI:** 10.21956/wellcomeopenres.23426.r86682

**Published:** 2024-07-15

**Authors:** Arun S. Kharat

**Affiliations:** 1 Laboratory of Applied Microbiology and Cancer Remedies, School of Life Sciences, Jawaharlal Nehru University, New Delhi, DELHI, India

A systematic analysis of antibiotics application and antimicrobial resistance is of paramount importance.  Due to diversity in term of recording, reporting and indiscriminate use of antimicrobials poses a great difficulty to ascertain exact AMR.  Introduction of GLASS by the WHO and focusing on selective pathogens under top priority, high priority with reference to antibiotic application/AMR has made it a lot easier to unfold AMR among eight pathogenic species.  Given that sporadic, epidemic infections contribute to AMR, which is by the several many species, beyond these 8 species covered under GLASS-WHO.  Industries those are involved in making/ disseminating antimicrobials have also been recording AMR but among a vast majority of species.  Both these approaches are working independently, authors in this article have taken a task of comparing Industrial and WHO GLASS dataset. Following are good points comes out of this article:

1. Combining Industrial data with WHO GLASS is definitely a value addition to estimate an AMR burden both in terms of bacterial species and country.  Such studies are more required to enhance quality of antimicrobial stewardship outcome OR strengthen the Antimicrobial Stewardship Program.
2. As there are not proper guidelines made available by the FDS/CLSI / EUCAST and in turn globally recognized body there exist a great amount of diversity in collecting and storing the data on AMR burden with reference to a country or a bacterial species. 
3. With meticulous approach authors have combined data and analyzed in the case of ATLAS (Pfizer) coverage is far better than other Industrial data for 17 years while for GLASS WHO just only for 4 years.  The SOAR (gsk) being the shortest 3 year from 9 countries on two pathogens.  Despite being there for 17 years ATLAS data covers 858,233 isolates from 83 countries, 345 bacterial species while just within 4 years The GLASS has data on 8 species from 71 countries and 11,855,726 bacterial isolates. It is worth noticing that WHO has highest intensity and makes better coverage. 
4. There is a whole lot of diversity in choosing bacterial species from Industrial data set, where rationale is very difficult to understand.  Several isolates reported from a bacterial species which not high in numbers, yet their incorporation in dataset adds to the global AMR. 
5. The take home message with a wonderful suggestion that there is a dire need to have a regulated guidelines which may be adopted both by the Global agency and Industrial sector.  With common guidelines, parameters comparison would be easier to draw a meaningful conclusion.  Despite being rich amount of data and burden the best possible conclusion at present could not be drawn due to fact that industrial dataset and WHO-GLASS do not follow same guidelines. 

Is the work clearly and accurately presented and does it cite the current literature?

Yes

If applicable, is the statistical analysis and its interpretation appropriate?

I cannot comment. A qualified statistician is required.

Are all the source data underlying the results available to ensure full reproducibility?

Partly

Is the study design appropriate and is the work technically sound?

Yes

Are the conclusions drawn adequately supported by the results?

Yes

Are sufficient details of methods and analysis provided to allow replication by others?

Yes

Reviewer Expertise:

Antimicrobial resistance, Clinical Microbiology, Environmental Microbology, Microbiome, Molecular Microbiology, anti-cancer

I confirm that I have read this submission and believe that I have an appropriate level of expertise to confirm that it is of an acceptable scientific standard.
